# Analysis of Honey and Environmental Samples from BEN Endemic Villages in Serbia: Identification of a Novel Human Exposure Pathway for Aristolochic Acids and Aristolactams

**DOI:** 10.1021/acs.jafc.5c06473

**Published:** 2025-06-20

**Authors:** Chun-Kit Au, Man-Lung Chin, Wing-Laam Luk, Ka-Wa Wong, Ling-Yung Che, Bi-Feng Yuan, Goran Ilić, Miljana Pavlović, Ho-Wai Chan, Jian Zhen Yu, Nikola M Pavlović, Zongwei Cai, Wan Chan

**Affiliations:** † Department of Chemistry, 58207 The Hong Kong University of Science and Technology, Clear Water Bay, Kowloon 999077, Hong Kong; ‡ Department of Occupational and Environmental Health, School of Public Health, Wuhan University, Department of Radiation and Medical Oncology, 12390 Zhongnan Hospital of Wuhan University, Wuhan 430071, China; § Institute of Forensic Medicine, Faculty of Medicine, University of Niš, Niš 18000, Serbia; ∥ Department of Anatomy, Faculty of Medicine, University of Niš, Niš 18000, Serbia; ⊥ Medical Faculty, University of Niš, Bulevar Dr Zorana D̵ind̵ića 81, Niš 18000, Serbia; # Innovation Center, University of Niš, Univerzitetski trg 2, Niš 18106, Serbia; ¶ Kidneya Therapeutics, Klare Cetkin 11, Belgrade 11070, Serbia; ∇ Eastern Institute of Technology Ningbo, Ningbo, Zhejiang 315200, China

**Keywords:** aristolochic acid, aristolactams, air pollution, biomass burning, Balkan endemic nephropathy

## Abstract

Dietary exposure to aristolochic acids (AAs) through AA-tainted flour is closely linked to the development of Balkan endemic nephropathy (BEN), a chronic kidney disease that is prevalent in rural farming villages in the Balkan region; however, additional exposure pathways would better explain the incidence rate of BEN. This study reveals for the first time that inhalation of AA-contaminated air, which often contains aristolactams (ALs)—genotoxic metabolites of AAs—represents an unrecognized exposure route. The presence of AAs was confirmed in local honey, and subsequent analysis of face masks worn by volunteers near flowering () weeds indicated that AAs may be airborne. Further investigation into the transport of AA-containing particles was conducted by analyzing outdoor residential surfaces (e.g., windowsills) in Serbia, detecting AA-I or AL-I in more than 20% of the samples, with concentrations ranging from 13 to 2470 pg and 1 to 8985 pg per 225 cm^2^, respectively. Additionally, it was found that burning generates particle-bound ALs. Given that weeds are often burned alongside wheat remnants for cooking, heating, and fertilizer production, these findings highlight airborne AAs and ALs as potentially key agents in the induction of BEN. In conjunction with the WHO’s notice that biomass burning significantly contributes to the high prevalence of respiratory diseases in the Balkans, this study identifies AAs and their analogs as air pollutants. Therefore, it is imperative to eliminate weeds from affected areas and to cease their use as heating and cooking fuel.

## Graphical abstract

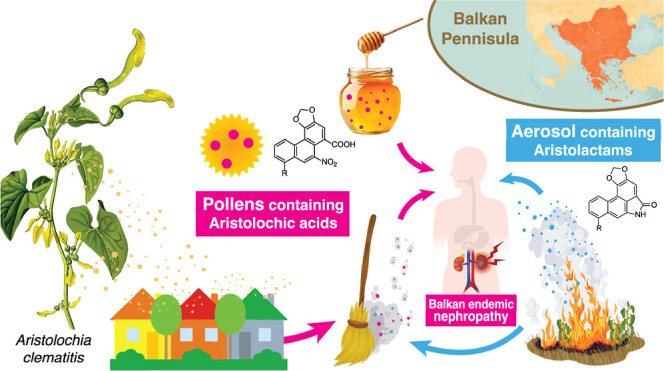

## Introduction

Balkan endemic nephropathy (BEN) is a chronic renal disease prevalent in rural farming communities along the tributaries of the Danube River in the Balkan Peninsula.^1,2^ Environmental factors such as ochratoxin, organic compounds leached from lignite, heavy metals, and viruses have been studied in relation to the disease. However, none of these factors is strongly supported by current evidence, and it is generally agreed that BEN is a multifactorial, familial condition.

Extensive research has established a link between BEN and the prolonged intake of aristolochic acids (AAs) from bread made with contaminated flour.^3–5^ This contamination is understood to occur via two mechanisms involving the growth of the weed (birthwort) in wheat fields: (i) seeds mix in during the wheat harvest^6^ and (ii) the uptake of AAs by the wheat crop from soil also contaminated by (Figure 1).^7,8^

**Figure 1:**
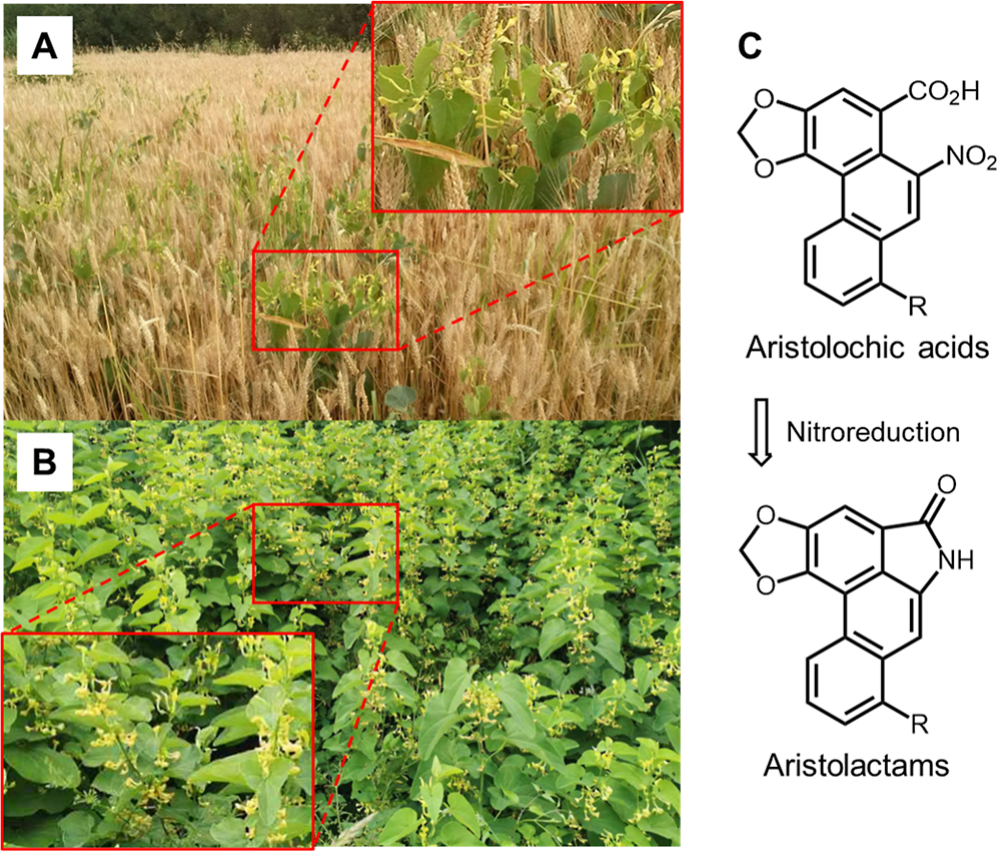
Photos of (A) a wheat field and (B) an abandoned field in Serbia, featuring weeds. Photos taken in Niš, Serbia in 2023. (C) The process of reducing aristolochic acids (AA-I, R = OCH_3_; AA-II, R = H) to form aristolactams (AL-I, R = OCH_3_; AL-II, R = H).

However, exposure to AAs is not limited to the ingestion of bread made with contaminated flour, but rather involves multiple environmental routes.^9–11^ For instance, AAs can transfer into groundwater and potentially affect a wider swath of the population (Figure 2).^9^ Moreover, AAs have been shown to survive on particle when grinding the plant parts of AA-containing species;^12–14^ although airborne transmission has not been further explored. Nevertheless, is a pollinating plant,^15^ and burning wheat remnants and weeds for cooking or heating is common practice in the Balkans,^16,17^ thus, representing two possible sources of AA-laden particles in the atmosphere.

**Figure 2:**
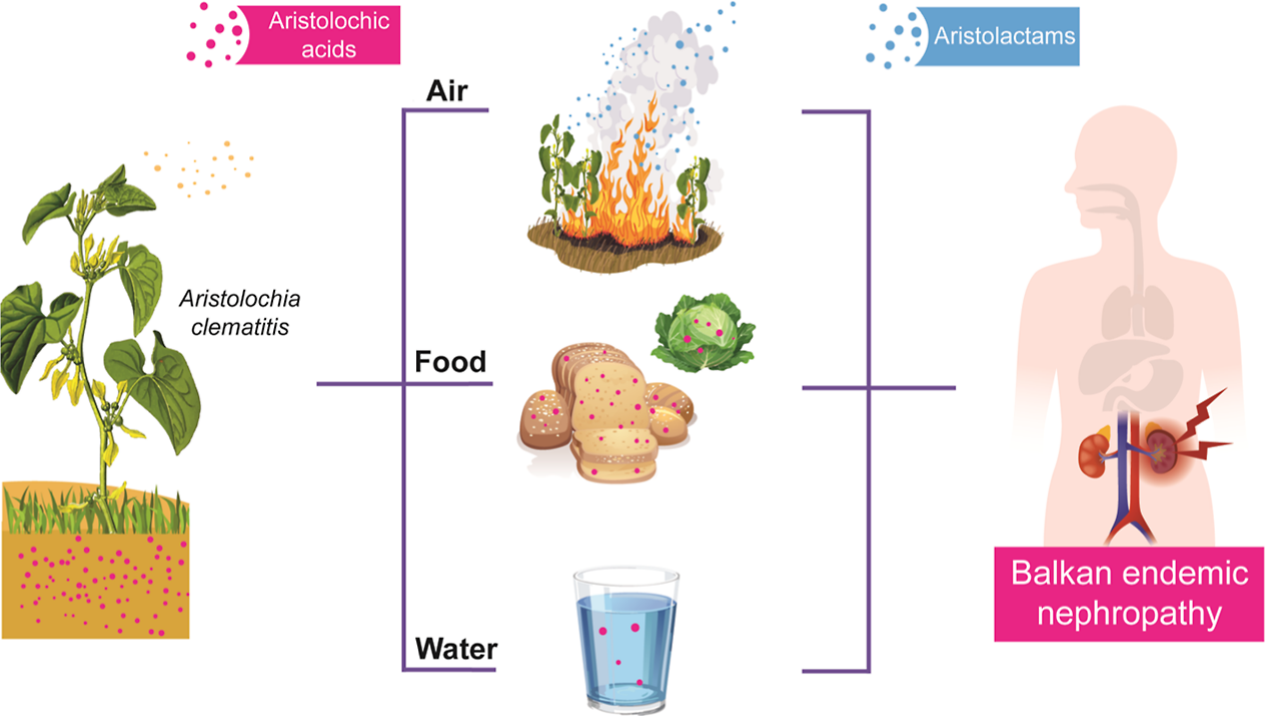
Pathways of human exposure to weed–derived aristolochic acids and aristolactams leading to Balkan endemic nephropathy.

To investigate these air transmission pathways for AAs, we quantified their amount in honey samples collected from wet markets, in face masks worn by volunteers, and in swabs of outdoor residential surfaces in Serbia. Many other pollutants, such as heavy metals and pesticides accumulate in honey,^18,19^ thus we suspected that AAs and their reduced product aristolactams (ALs) may be found in the honey produced from the region. In order to achieve this, we developed a method for extracting and quantifying the major components of the AAs family—aristolochic acid I (AA-I), aristolochic acid II (AA-II), aristolactam I (AL-I), and aristolactam II (AL-II)—in honey using liquid chromatography coupled with tandem mass spectrometry (LC–MS/MS).

We then assessed the potential transmission of these pollutants into ambient environments. We extracted and analyzed the particles collected with a swab test method and on face masks—known to be excellent pollen-trapping devices^20^—worn by volunteers during the sampling process. Additionally, experiments were conducted to test the potential release of AAs and/or ALs into the air when plants containing these compounds are burned. AAs and/or ALs were detected for the first time in honey, face masks, alcohol swab, and air filter samples. These findings provide the first evidence of the airborne transportation of AAs and/or ALs via combustion-derived fine particles and aerosols in the affected areas, highlighting the urgent need for attention from both the public and regulatory agencies.

## Materials and Methods

### Chemicals and Material

Aristolochic acid I was purchased from Acros Organics (Morris Plains, NJ), while aristolochic acid II and benz­[*cd*]­indol-2­(1*H*)-one were obtained from Sigma-Aldrich (St. Louis, MO). Aristolactam I and aristolactam II were synthesized from AA-I and AA-II based on a previous study.^21^ plants were collected from cultivation fields in Serbia, and herbal plants (, , , and ; TABLE S1) were purchased online. Methanol and acetonitrile were obtained from Tedia (Fairfield, OH). Ultrapure reagent water was produced using a Pall Cascada laboratory water purification system (Port Washington, NY).

### Alcohol Swab Sample Collection and Extraction

Alcohol swab samples (*n* = 261) were collected from various outdoor locations in four villages and the city of Niš, Serbia, during June 2023 and July 2024. Sampling sites included fence tops, window frames, mailboxes, and similar surfaces. Each swab was applied to an area approximately 15 × 15 cm^2^, then sealed in a polypropylene bag and sent to the laboratory for analysis. Prior to analysis, each swab was placed in a 2 mL polypropylene tube, to which 1 mL of extraction solvent (methanol/water/acetic acid; 70/25/5) was added. The mixture was sonicated at room temperature for 30 min. Subsequently, 10 μL of benz­[*cd*]­indol-2­(1*H*)-one (10 μg/L) was added to 90 μL of the extract before analysis by LC–MS/MS.

### Honey Sample Collection and Extraction

Honey samples (*n* = 42) were collected from two weekend markets in Niš, Serbia, concurrently with the alcohol swab samples. The samples were stored at room temperature until analysis. Prior to analysis, AAs and ALs were extracted by salt-out method.^22^ In brief, 1 g of each honey sample was accurately weighed in polypropylene tube containing 2 g sodium chloride, and dissolved in 5 mL of 5% acetic acid aqueous solution. The samples were then added with 3 mL of acetonitrile and shaken for 30 min. After centrifugation, the organic layer was collected and dried under a nitrogen stream. The residue was redissolved in 0.5 mL of extraction solvent for cleanup via solid-phase extraction (SPE; Grace, High-flow C18, 500 mg), as previously described.^7,8,10^ The organic eluate from the SPE column was dried under a nitrogen stream, and the residue was redissolved in 50 μL of 70% methanol containing 1 μg/L benz­[*cd*]­indol-2­(1*H*)-one for LC–MS/MS analysis.

### Face Mask Sample Collection and Extraction

Face mask samples were collected by volunteers during the flowering season of in the village Brestovac in June 2023. An exposure duration-dependent accumulation of AAs and ALs was assessed by volunteers (*n* = 3) wearing polypropylene face masks for 0, 10, 20, and 30 min at a distance 0.5 m away from the weed. Similarly, the distance-dependent accumulation of AAs and ALs trapped on masks was evaluated by wearing polypropylene face masks for 30 min, at a distance of 0.5, 1.5, and 3 m away from the weed.

The worn masks were sent back to laboratory for analysis. Half of each mask sample was cut into 1 cm^2^ pieces, added with 10 mL extraction solvent, and sonicated at room temperature for 30 min. One milliliter of the sample aliquot was processed for SPE cleanup as per the outlined procedure. The SPE eluate was dried under stream of nitrogen gas, and the residue was redissolved in 50 μL of 70% MeOH containing 1 μg/L benz­[*cd*]­indol-2­(1*H*)-one for LC–MS/MS analysis. Field blanks were prepared using unused masks from the same batch that underwent the same transportation and sample preparation processes, but without air sampling.

### Burning Experiment

To test for the potential release of AAs from burning weed, plants were collected from wheat/maize fields in Serbia, sun-dried, mixed with approximately ten times their weight in wheat remnants, and burned in a typical field setting. The smoke, containing both aerosols and soot, was collected at a flow rate of 3 L/min onto a PTFE filter. The filter was then extracted using 3 mL of extraction solvent, processed with SPE, dried under nitrogen, and redissolved in 200 μL of 70% MeOH containing 1 μg/L benz­[*cd*]­indol-2­(1*H*)-one for LC–MS/MS analysis. Filters collected from the burning of wheat remnants alone served as a control.

Similarly, herbal plants (, , , and ) containing varying amounts of AAs and ALs (Table S1) were mixed with ten times their weight in wheat remnants and burned inside a fume hood. The generated soot and aerosols were collected and analyzed using methods similar to those described above. As a positive control, samples were collected from burning a similar amount of wheat remnants spiked with 10, 50, and 200 mg/kg of AA-I or 1, 5, 10, and 20 mg/kg of AL-I dissolved in methanol (*n* = 3), whereas wheat remnants spiked with methanol only was used as the negative control.

### LC–MS/MS Analysis

LC–MS/MS analysis of AAs and ALs was performed essentially as described previously, with modifications.^10,23,24^ In brief, a Waters Acquity UPLC (Waters; Milford, MA) coupled with a Waters TQ-XS triple quadrupole LC–MS/MS system equipped with an electrospray ionization (ESI) interface was used. Ten microliters of the samples were injected into a Roc C18 column (100 × 3 mm, 5 μm; Restek; Bellefonte, PA) maintained at 40 °C. The column was eluted with a binary solvent mixture of solvent A (13 mM ammonium acetate buffer, pH 4.2) and solvent B (acetonitrile) at a constant flow rate of 0.5 mL/min, following this gradient: 0 to 1 min, 10% B; 1 to 8.5 min, linearly ramped to 75% B; 8.6 min, 100% B; held for 2 min before reconditioning.

MS data were acquired in positive ESI mode with optimized source parameters: ionspray voltage of 1500 V, cone energy of 20 V, and nebulizer gas flow and temperature set to 1000 L/min and 550 °C, respectively. The MS operated in multiple reaction monitoring (MRM) mode, utilizing the following MRM transitions: *m*/*z* 359/298, *m*/*z* 329/268, *m*/*z* 294/279, and *m*/*z* 264/151 for quantitative analysis of AA-I, AA-II, AL-I and AL-II, respectively; *m*/*z* 359/296, *m*/*z* 329/294, *m*/*z* 294/251, and *m*/*z* 264/206 for qualitative analysis of AA-I, AA-II, AL-I and AL-II, respectively (Figure S1). The internal standard, benz­[*cd*]­indol-2­(1*H*)-one, was monitored at *m*/*z* 170/115.

## Results and Discusion

### Honey Sample Analysis

Prior studies have identified various phytochemicals, including grayanotoxins, triptolides, tutin, and pyrrolizidine alkaloids, in honey.^25,26^ Despite not yet reported in the literature, there was a suspection that honey produced from the area might also contain AAs. To investigate this, we collected honey samples from weekend markets in Niš, Serbia, and analyzed them using our well-validated method (Table S2).

Among the 42 honey samples tested, AA-I was detected in 3 samples, with 2 of these exceeding the minimum quantification limit (143 pg/g), at concentrations ranging from 222 pg/g to 390 pg/g. In contrast, AA-II, AL-I, and AL-II were not detected in any of the samples, probably attributed to their lower concentration than that of AA-I as has been reported from an seed analysis.^24,27,28^ Here, it is worth mentioning that AA-I is the most carcinogenic member of the AA family.^3,4,29^

The low detection rate of AAs in honey samples is probably attributed to the fact that the flower of is relative small in general and maybe difficult for the bees to reach. It is also possible that the contaminants arose from pollen of other non- flowering plants of the area which have updated AAs from the environment. Nevertheless, this is the first instance of detecting AAs in honey samples. Figure S2 shows a typical chromatogram obtained from LC–MS/MS analysis of AA-I in one of the honey samples.

### Detection of AAs and ALs on Face Masks Used near 

After detecting AA-I in some of the collected honey samples, we further validated this hypothesis by testing face masks worn by three volunteers standing downwind from blooming weeds in an abandoned field in Serbia on a windy day. Here face masks were used as pollen-collecting devices because face masks have a high pollen trapping efficiency (>99%) and have been demonstrated as personal exposure dosimeters for airborne pollutants in other studies.^20,30–35^

While we did not detect any AAs or ALs in the field blank samples, we found that AA-I was present in all face masks worn by volunteers positioned at three different distances downwind from the weeds. For the same reasons as in the honey analysis, AA-II, AL-I, and AL-II were not detected in any of the samples. Figure S3 shows a typical chromatogram obtained from LC–MS/MS analysis of AA-I in one of the mask samples. Furthermore, a clear exponentially decreasing trend of distance-dependent accumulation of AA-I on the masks was observed (Figure 3A), with the highest amount of AA-I detected in samples collected from 0.5 m away from the weeds.

**Figure 3:**
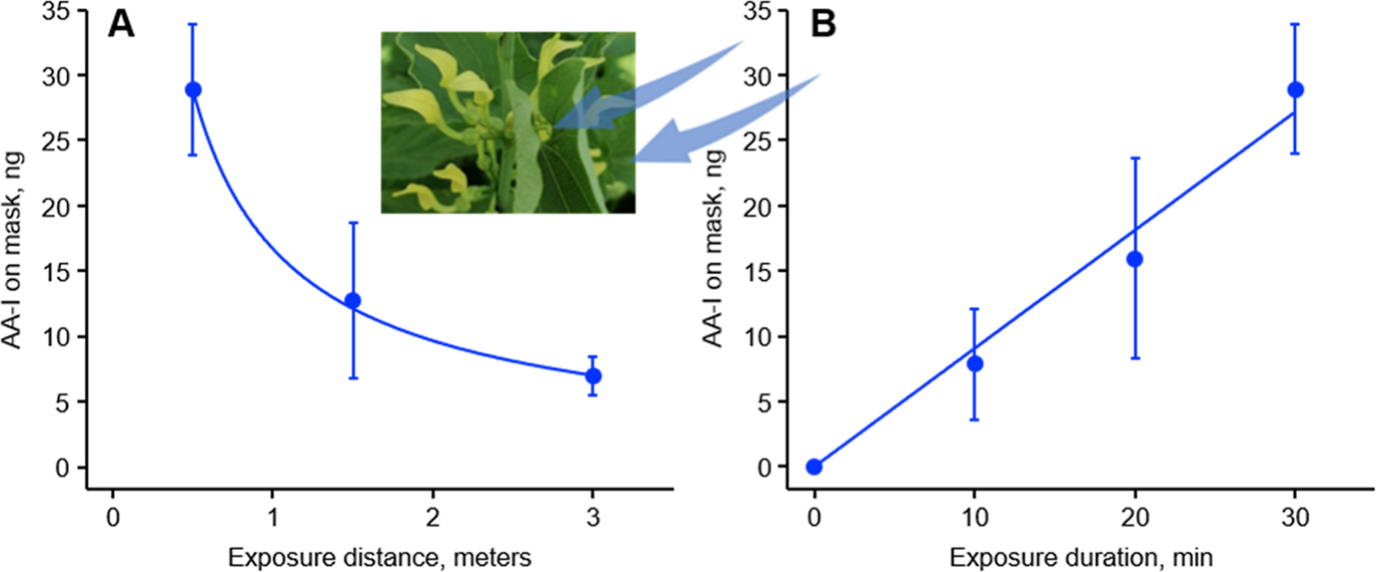
Amount of AA-I trapped on face masks worn by volunteers at (A) different distances away from the blooming weeds and (B) for different durations while standing 0.5 m away from the blooming weeds in a cultivation field in Serbia. Fitting the data to a power law curve and by linear regression yielded equations of = 16.7*x* ^–0.79^ (*r* ^2^ = 0.99) and *y* = 0.90*x* (*r* ^2^ = 0.99), respectively. The data represent mean ± SD for three independent experiments. Shown in the inset of panel A is a picture taken in June 2023 showing the flower of .

Analysis of the masks worn by volunteers for different duration standing at 0.5 m away from the weeds revealed an exposure duration-dependent accumulation of AA-I on the masks. Together with the observation that AAs were not detected in mask samples worn by volunteers at upwind from the weeds, these results indicate that some pollen is suspended in the air and may be carried by the wind over distances.

### Analysis of Alcohol Swabs for AAs and ALs

Having demonstrated the potential presence of AAs in the pollen of using face masks, we proceeded to test for the transport of AA-containing particles, including pollen, by collecting dust samples using alcohol swabs from outdoor residential surfaces in four villages and the city of Niš in Serbia,^19,36,37^ regions significantly affected by BEN. To minimize contamination from footwear, we avoided sampling dust on walkways.

Among the 261 alcohol swab samples tested, AA-I, AA-II, AL-I, and AL-II were detected in 60, 5, 12, and 3 samples, respectively, at levels measured in picograms per swab (Table 1). Notably, the highest amount of AA-I (2470 pg) was detected in a sample collected from a window frame adjacent to flowering plants. This finding provides additional evidence that pollen may serve as a medium for transmitting AAs through the air. Figure 4 shows typical chromatograms obtained from analyzing AA-I, AA-II, AL-I, and AL-II in one of the alcohol swab samples.

**Table 1:** Amounts of Aristolochic Acids and Aristolactams in Alcohol Swab (*n* = 261) Collected from Outdoor Surfaces in Villages and in Niš, Serbia

	AA-I	AA-II	AL-I	AL-II
no. of samples analyzed	261			
no. of positive sample^a^	60	5	14	5
amount range, pg–pg^b^	13–2470	47–637	1–8985	2–942
average amount, pg^b^	147	348	748	239

a Samples with amount above the limit of detection (LOD): 8.1 pg AA-I, 28.3 pg AA-II, 0.6 pg AL-I, and 1.3 pg AL-II.

b Samples with AAs and ALs amount below the limit of quantitation (LOQ) were assigned an amount of one-half of the LOQ: 13.4 pg AA-I, 46.7 pg AA-II, 0.99 pg AL-I, and 2.1 pg AL-II.

**Figure 4:**
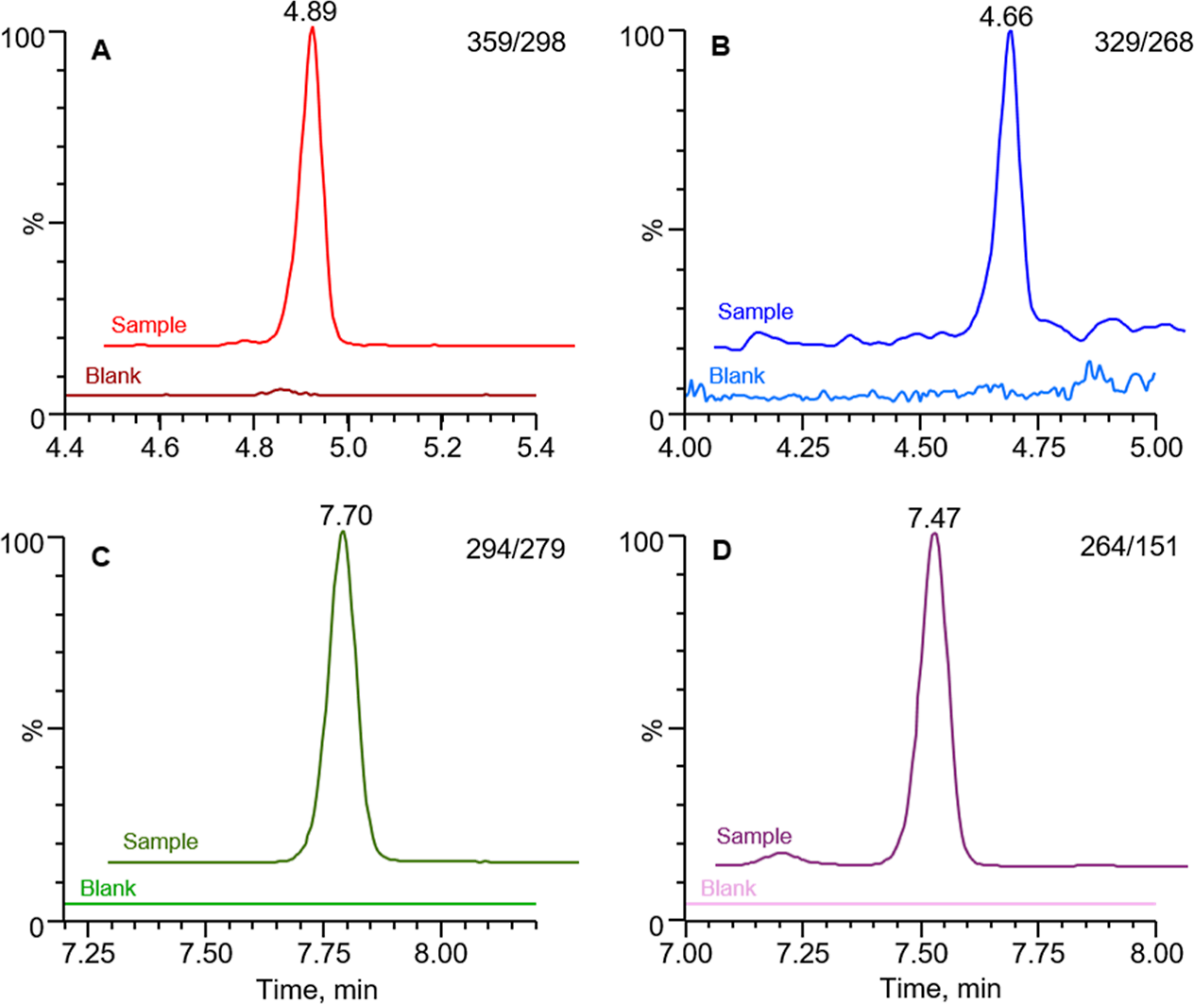
Reconstructed chromatograms from LC–MS/MS analysis of aristolochic acid I (AA-I); (A), aristolochic acid II (AA-II); (B), aristolactam I (AL-I); (C), and aristolactam II (AL-II); (D) in an alcohol swab sample collected from typical outdoor residential surfaces in Serbia, along with a field blank for comparison.

Despite the absence in honey and mask samples, the high detection frequencies of both AL-I and AL-II in the collected dust samples are intriguing, suggesting that there may be alternative, nonpollen sources contributing to the detected signals of AAs and ALs. Further investigation is warranted to identify these sources and assess their potential implications for public health and environmental exposure.

### Investigation of Airborne AAs and ALs from Burning 

As previously discussed, the high frequencies of detecting ALs (AL-I and AL-II) on outdoor residential surfaces highlight alternative pollution sources for AAs and ALs. Due to the extensive practice of burning wheat remnants for cooking, heating,^16,17^ and as fertilizer in situ, we investigated the potential for weeds, commonly found in these fields, to release AAs and/or ALs into the environment when burned. This is particularly relevant given that the burning of both wheat remnants and would occur simultaneously if effort was not taken to avoid contamination during harvesting or before burning. Despite the high boiling points of AAs (over 600 °C) and ALs (over 400 °C), as well as the unknown reactions that may occur during combustion, we aimed to test whether the burning process could still release these compounds into the environment, either through soot or as aerosols.

We detected AL-I in air samples collected for the first time from the burning of wheat remnants mixed with weeds (Figure S4). Additionally, there was a clear relationship between the amount of burned and the accumulation of AL-I (Figure 5). Although AAs were present in much higher concentrations in the weed, they were not detected on filters. This is likely due to their much higher boiling points compared to the temperatures reached during the burning, which can go up to 400 °C —close to the boiling points for ALs. As a result, some of the AL-I may have became airborne during the burning process and quickly recondensed onto other coemitted particles.^38^ It is also important to note that AA-II and AL-II were found in much lower concentrations than AA-I and AL-I in , and AL-II was not detected in any of the samples. These results provided evidence that the ALs detected in the above stated alcohol swabs test may have derived from burning particles.

**Figure 5:**
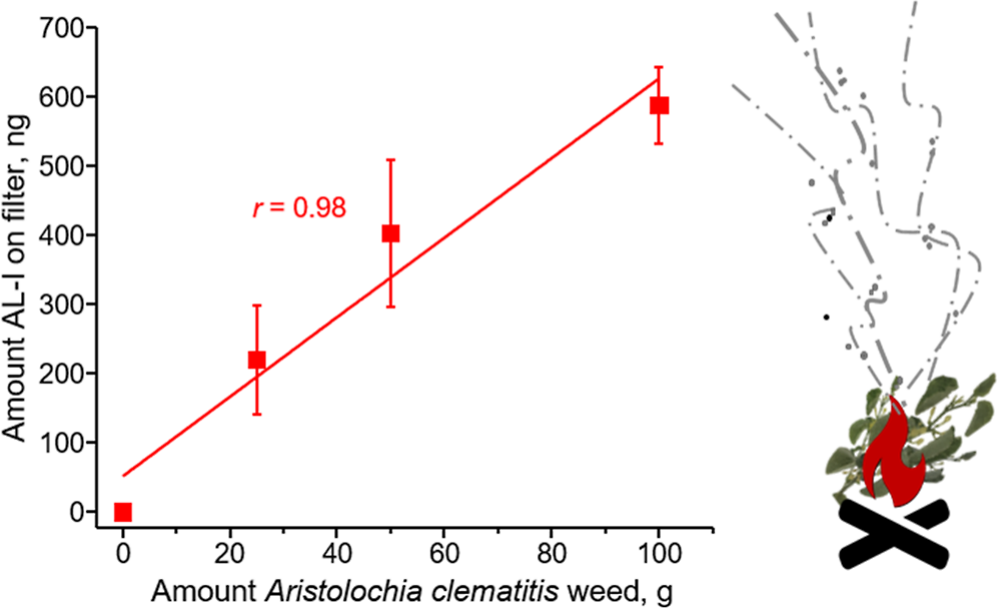
Correlation of the amounts of aristolactam I (AL-I) on air filter samples with the amount of weed burned with wheat remnants in a typical cultivation field in Serbia. Air samples were collected on a PTFE filter positioned 70 cm above the flame for analysis. The data are presented as mean ± SD from three independent experiments.

Although AL-I are in general less genotoxic than AA-I, its detection in air samples is particularly concerning. Previous studies have shown that the cellular absorption of AL-I is at 900 times higher than AA-I, with similar cytotoxicity, and can form the same DNA adducts upon metabolic activation.^39,40^ Notably, if condensed on submicron particles, airborne AL-I may enter the lungs directly through inhalation, bypassing the first-pass effect of the liver that occurs with ingestion, which may further enhance their toxicity.^41,42^

### Source of AL-I in the Air

There are two possible sources for the AL-I detected in the burning emissions. First, some native AL-I may have vaporized from the during the burning process. Second, due to the presence of reducing elements such as Fe and Cu in the wheat remnants and , it is possible that some AA-I was reduced to AL-I during burning and subsequently vaporized (Figure 1).^43^ We have demonstrated in our previous study that transition metals such as Fe are highly efficient in reducing AAs to ALs.^21^

To investigate the potential sources of AL-I in the burning fumes, we first determined the concentrations of AA-I and AL-I in various herbal plants (Table S1). These AA- and AL-containing plants were mixed and burned together with wheat remnants in the same manner as the weeds. The amounts of AL-I in air samples from the burning were then correlated with the amounts of AA-I and AL-I in the burned herbs.

Interestingly, the analysis revealed an excellent correlation between the amounts of AL-I in the collected filter samples and the concentrations of both AA-I and AL-I in the herbs burned (*r* > 0.97; Figure 6A). This result indicates that, in addition to being produced from the vaporization of native AL-I, AL-I may also be generated from AA-I during the burning process. The hypothesis that airborne AL-I originated from the evaporation of native AL-I and from the reduction of AA-I during burning was further supported by the detection of AL-I in the burning fumes of AL-I- or AA-I-spiked wheat remnants (Figure 6B), in which a close correlation of AL-I collected on the filter samples with that of the amount of both AA-I and AL-I spiked into the wheat remnants.

**Figure 6:**
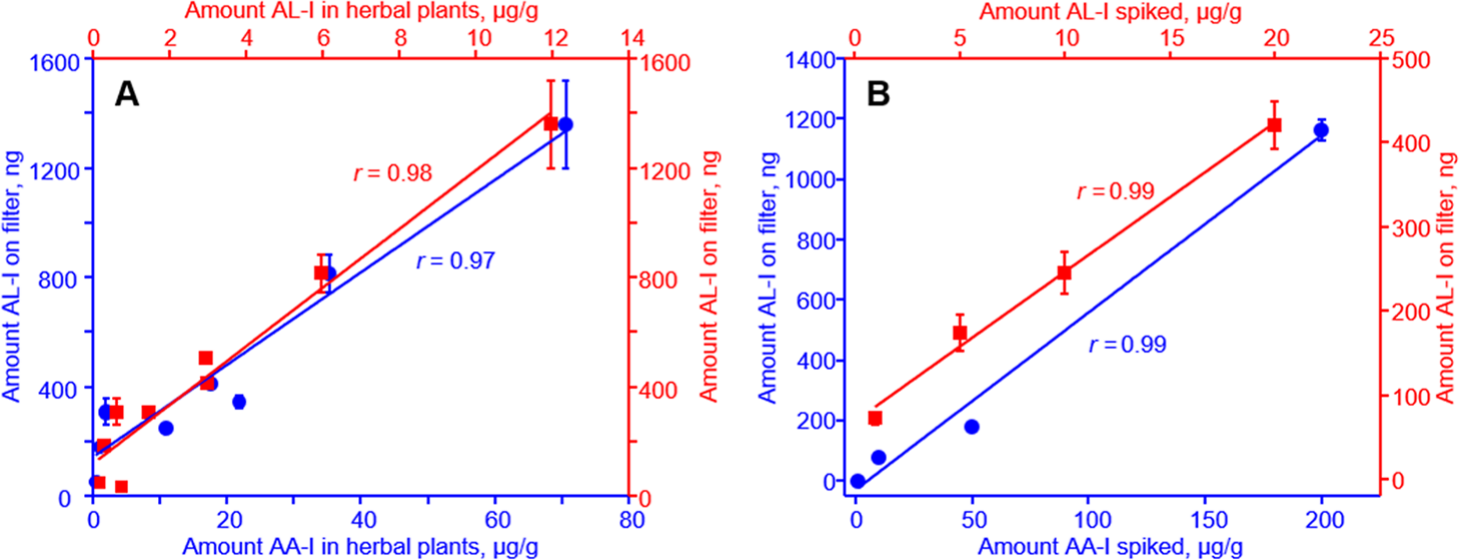
Correlation of the amounts of AL-I collected on PTFE filters with the amounts of AA-I and AL-I (A) in various AA- and AL-containing herbs and (B) in AA-I- or AL-I-spiked wheat remnants burned in-house as described in Materials and Methods. Shown in the plots are Pearson correlation coefficients of the analyses. The data represent mean ± SD for three independent experiments.

The possibility that some of the detected AL-I was generated through the in-source reduction of AA-I mediated by reactive metals was further validated by burning weed spiked with varying amounts of iron­(II) sulfate. An iron­(II) concentration-dependent accumulation of AL-I trapped on the filter paper was observed (Figure 7). Given that iron is the most abundant metal species in plants, it is plausible that some AAs may be reduced by Fe to form ALs, which could then vaporize in the high-temperature flame before recondensing on to biomass burning particles.

**Figure 7:**
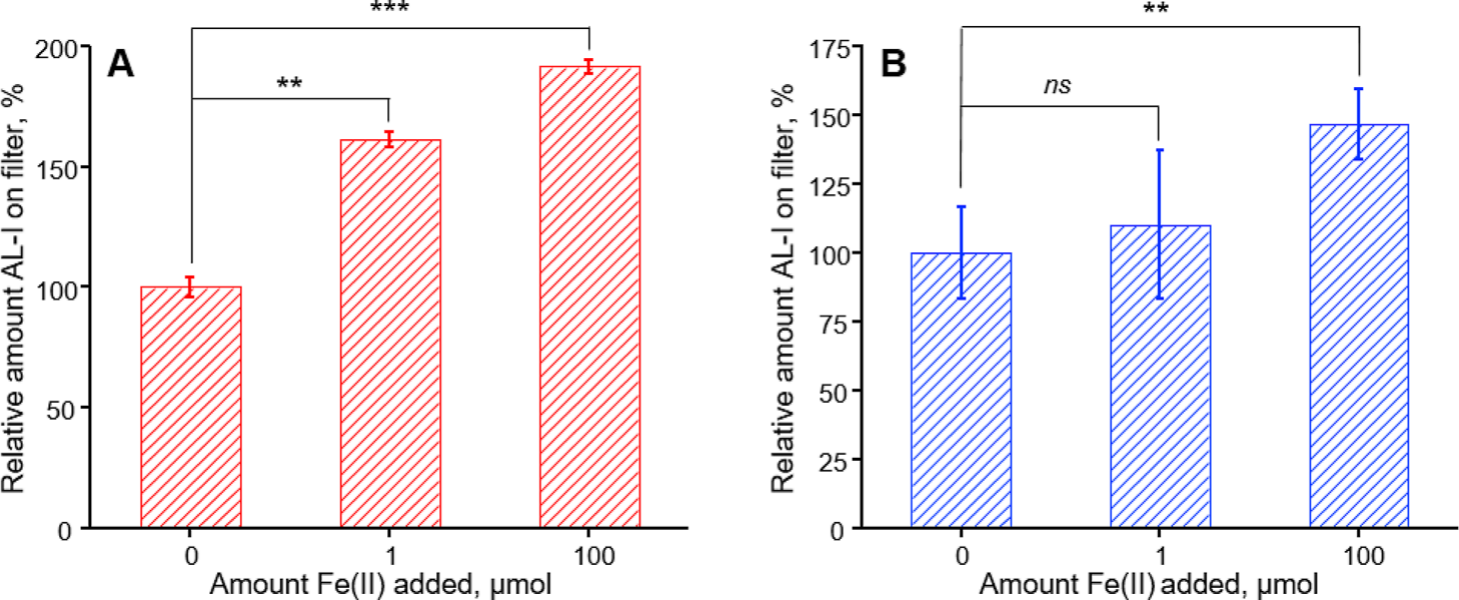
Fe­(II) concentration-dependent accumulation of AL-I trapped on the air filter samples from burning (A) AA-I-spiked wheat remnants and (B) 5 g added with different amounts of Fe­(II) sulfate. Air samples were collected on a PTFE filter positioned 70 cm above the flame for analysis in-house. The data represent mean ± SD for three independent experiments. Statistical significances were compared against control group by Student’s *t*-test, and were indicated by asterisks (ns, no significant difference; **, *p* ≤ 0.01; and ***, *p* ≤ 0.001).

An attempt was made to determine whether the detected AL-I was present in gaseous or particulate/aerosol form. To achieve this, we conducted air sampling using a series connection of PTFE and PUF filters, which are designed to trap AL-I in particulate/aerosol and gaseous forms, respectively. Upon separate analysis of the filters, the majority of the signals were detected in the PTFE filter, while signals were not detectable in the PUF filter. This indicates that most of the AL-I was present in particulate or aerosol form during the sampling process.

## Implications

By analyzing honey, alcohol swabs, and masks worn by volunteers in rural farming villages in Serbia, this study reveals that, in addition to the previously known exposure pathway through contaminated food and water, humans are also inadvertently exposed to AAs and their analogues through contaminated air. This exposure may contribute to chronic kidney disease and respiratory tract diseases among residents of the Balkan Peninsula. Prolonged exposure through inhalation of AA-containing pollen from weeds, as well as aerosols and soot emitted from burning these weeds, likely represents an unrecognized exposure pathway that increases the risk of developing BEN and respiratory diseases. Given that the AA-producing weed is widespread in the Balkan region, its pollen is also prevalent. Furthermore, the weed is often mixed with and burned alongside wheat remnants for cooking, heating, and as fertilizer in affected areas, making it a significant source of environmental AAs and ALs. Recent calls from the World Health Organization (WHO) for action on the public health risks associated with AAs underscore the importance of our findings. Our results highlight that exposure to AAs and ALs through inhalation may pose a health hazard that warrants the attention of both the general public and regulatory agencies. It is essential that AAs, particularly ALs, be included in future air monitoring programs in affected areas.

## Supplementary Material



## Notes

Concentrations of AAs and ALs in herbal plants used in burning experiment. Method recoveries and detection limits of AAs and ALs in honey and environmental samples. Typical chromatograms obtained from LC–MS/MS analysis of AA-I in a honey sample and mask sample, and AL-I in an air filter (PDF)
