# Supplementary material for: Analysis of Honey and Environmental Samples from BEN Endemic Villages in Serbia: Identification of a Novel Human Exposure Pathway for Aristolochic Acids and Aristolactams
Source: J Agric Food Chem. 2025 Jun 20;73(26):16293–300. doi: 10.1021/acs.jafc.5c06473 (PMC12322912; doi:10.1021/acs.jafc.5c06473)
Supplement: Supplementary file 1 [file jf5c06473_si_001.pdf]

Supporting Information

Analysis of Honey and Environmental Samples from BEN Endemic Villages in Serbia: Identification of a Novel Human Exposure Pathway for Aristolochic Acids and Aristolactams

Chun-Kit Au [a,#], Man-Lung Chin [a,#], Wing-Laam Luk [a], Ka-Wa Wong [a], Ling-Yung Che [a], Bi-Feng Yuan [b], Goran Ilić [c], Miljana Pavlović [d], Ho-Wai Chan [a], Jian Zhen Yu [a], Nikola M. Pavlović [e,f,g,*], Zongwei Cai [h], Wan Chan [a,*]

[a] Department of Chemistry, The Hong Kong University of Science and Technology, Clear Water Bay, Kowloon 999077, Hong Kong

[b] Department of Occupational and Environmental Health, School of Public Health, Wuhan University, Department of Radiation and Medical Oncology, Zhongnan Hospital of Wuhan University, Wuhan 430071, China

[c] Institute of Forensic Medicine, Faculty of Medicine, University of Niš, Niš 18000, Serbia

[d] Department of Anatomy, Faculty of Medicine, University of Niš, Niš 18000, Serbia

[e] Medical Faculty, University of Niš, Bulevar Dr Zorana Đinđića 81, Niš 18000, Serbia

[f] Innovation Center, University of Niš, Univerzitetski trg 2, Niš 18106, Serbia

[g] Kidneya Therapeutics, Klare Cetkin 11, Belgrade 11070, Serbia

[h] Eastern Institute of Technology Ningbo, Ningbo, Zhejiang 315200, China

[#] the authors contributed equally to this work.

[*] Corresponding authors: chanwan@ust.hk, nikpavster@gmail.com

TABLE OF CONTENTS

TABLE S1. Concentrations of aristolochic acids and aristolactams in herbal plants used in the study. (*Page S3*)

TABLE S2. Method recovery and detection limits of aristolochic acid and aristolactams in honey, face masks, alcohol swab, and PTFE filter samples. (*Page S4*)

Figure S1. Typical LC−MS/MS chromatogram of aristolochic acid I, aristolochic acid II, aristolactam I, and aristolactam II in a standard solution. (*Page S5)*

Figure S2. Typical LC−MS/MS chromatogram of aristolochic acid I in a honey sample. (*Page S6)*

Figure S3. Typical LC−MS/MS chromatogram of aristolochic acid I in a mask sample. (*Page S6*)

Figure S4. Typical LC−MS/MS chromatogram of aristolactam I in an air filter sample. (*Page S7*)

REFERENCES (*Page S7*)

TABLE S1. Concentrations of aristolochic acids and aristolactams in herbal plants determined using a reduction column coupled LC–FLD method.

	AA-I, mg/kg	AA-II, mg/kg	AL-I, mg/kg	AL-II, mg/kg
Asari Radix et Rhizoma	21 ± 3.2	ND[a]	14.8 ± 2.7	ND
Herba Aristolochiae	10.3 ± 1.8	1.4 ± 0.4	3.2 ± 0.3	ND
Fructus Aristolochiae	352.5 ± 13.9	32.9 ± 3.2	59.7 ± 2.4	ND
Herba Aristolochiae Mollissimae	218.7 ± 36.2	11.3 ± 0.5	ND	ND

[a]ND signified not detected. Method detection limits for AA-I, AA-II, AL-I, and AL-II are 5.0 µg/kg, 6.6 µg/kg, 4.5 µg/kg, and 6.3 µg/kg, respectively.

TABLE S2. Method recovery and detection limits of detecting AA-I, AA-II, AL-I, and AL-II in honey, face masks, alcohol swab, and PTFE filter samples.

	**Amount spike**	**[a]Amount found**	**[a]Recovery, %**	**Method detection limit**, pg
**[b]*Honey***				
**AA-I**	2.0 ng	1.2 ± 0.1 ng	59.7 ± 10.1	41.2
	10.0 ng	5.8 ± 0.6 ng	57.7 ± 10.6	
**AA-II**	2.0 ng	1.4 ± 0.1 ng	68.3 ± 9.3	101.0
	10.0 ng	6.6 ± 0.5 ng	66.3 ± 7.7	
**AL-I**	2.0 ng	1.5 ± 0.2 ng	73.6 ± 15.9	12.6
	10.0 ng	7.7 ± 0.1 ng	77.4 ± 1.6	
**AL-II**	2.0 ng	1.5 ± 0.2 ng	74.2 ± 11.7	86.8
	10.0 ng	7.9 ± 0.5 ng	78.7 ± 6.2	
Face mask				
**AA-I**	50.0 pg	32.7 ± 2.2 pg	65.4 ± 6.8	7.8
	200.0 pg	117.9 ± 5.1 pg	59.0 ± 4.3	
**AA-II**	50.0 pg	25.0 ± 3.0 pg	50.0 ± 12.1	5.7
	200.0 pg	103.6 ± 12.1 pg	51.8 ± 11.7	
Alcohol swab				
**AA-I**	250.0 pg	161.1 ± 4.1 pg	64.5 ± 2.6	8.1
	500.0 pg	307.2 ± 15.2 pg	61.4 ± 5.0	
**AA-II**	250.0 pg	227.1 ± 23.8 pg	90.8 ± 10.5	28.3
	500.0 pg	453.6 ± 10.6 pg	90.7 ± 2.3	
**AL-I**	10.0 pg	4.4 ± 0.3 pg	44.3 ± 7.3	0.6
	20.0 pg	8.8 ± 1.1 pg	44.1 ± 12.4	
**AL-II**	10.0 pg	5.4 ± 0.5 pg	54.0 ± 9.6	1.3
	20.0 pg	9.6 ± 1.2 pg	48.1 ± 12.5	
**[b]*PTFE filter***				
**AA-I**	1.0 ng	0.9 ± 0.1 ng	87.2 ± 13.1	54.1
	5.0 ng	4.3 ± 0.4 ng	86.2 ± 10.1	
**AA-II**	1.0 ng	0.9 ± 0.1 ng	92.2 ± 13.1	416.0
	5.0 ng	4.3 ± 0.3 ng	86.1 ± 13.1	
**AL-I**	1.0 ng	0.7 ± 0.1 ng	70.5 ± 4.4	93.6
	5.0 ng	3.7 ± 0.8 ng	73.5 ± 20.5	
**AL-II**	1.0 ng	0.9 ± 0.2 ng	85.7 ± 18.8	370.0
	5.0 ng	3.5 ± 0.7 ng	70.2 ± 19.0	

[a] The data represent mean ± SD for three independent experiments

[b]The analyses were conducted on a Waters Acquity UPLC coupled with an AB Sciex API 4000 QTRAP LC−MS/MS system, as reported previously.[1]

Figure S1. Typical LC−MS/MS chromatogram of aristolochic acid I, aristolochic acid II, aristolactam I, and aristolactam II in a standard solution mixture.

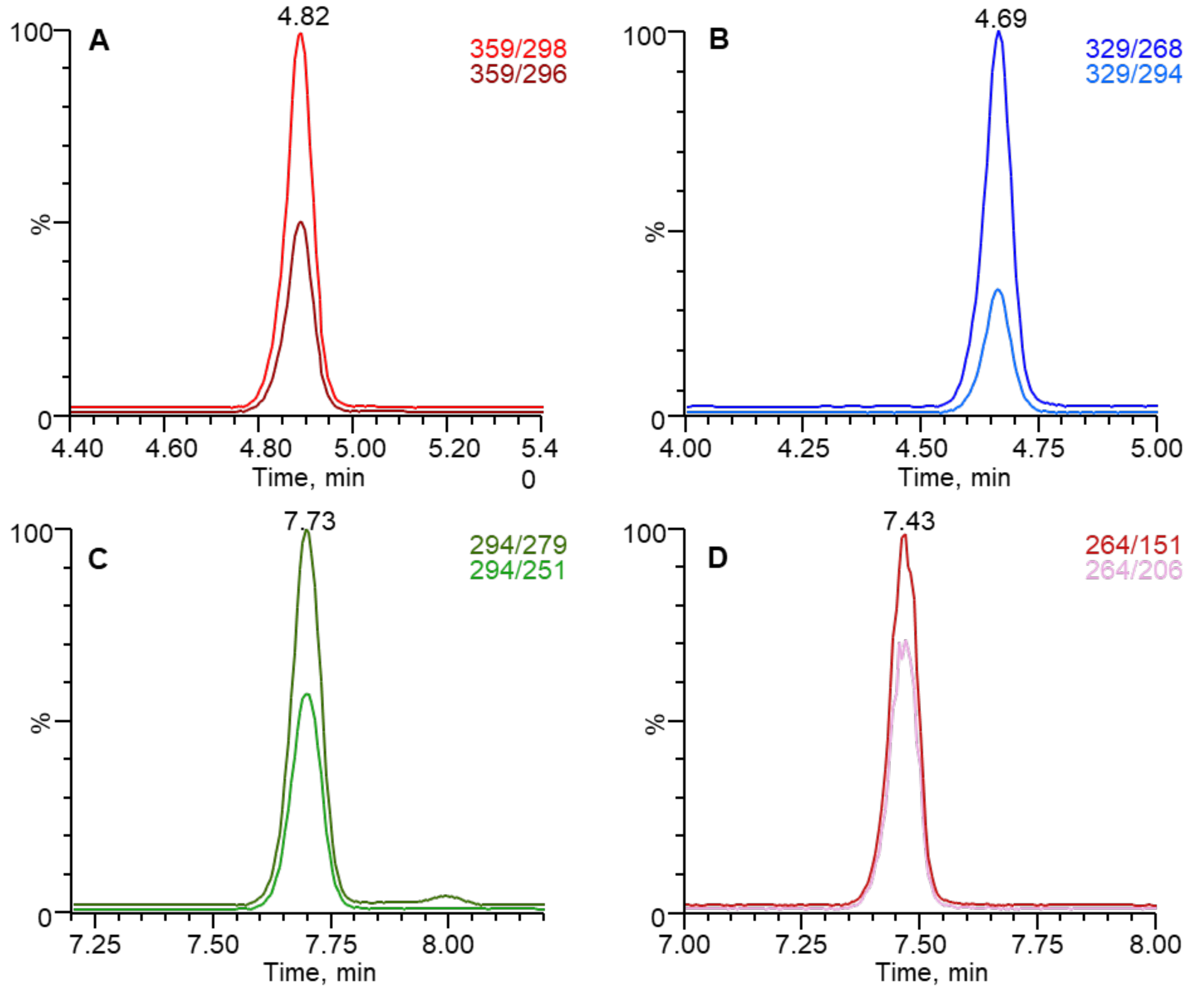

Analysis performed on a Waters TQ-XS triple quadrupole LC-MS/MS system.

Figure S2. Typical LC−MS/MS chromatogram of aristolochic acid I in (A) standard solution and (B) a honey sample.

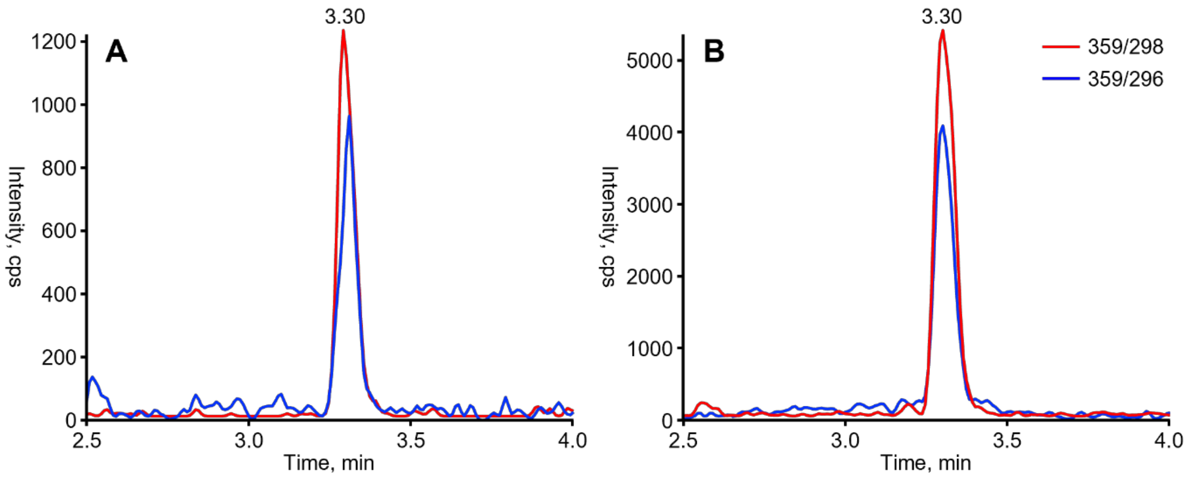

Analysis performed on a Sciex API 4000 QTRAP LC-MS/MS system.

Figure S3. Typical LC−MS/MS chromatogram of aristolochic acid I in (A) standard solution and (B) a mask sample.

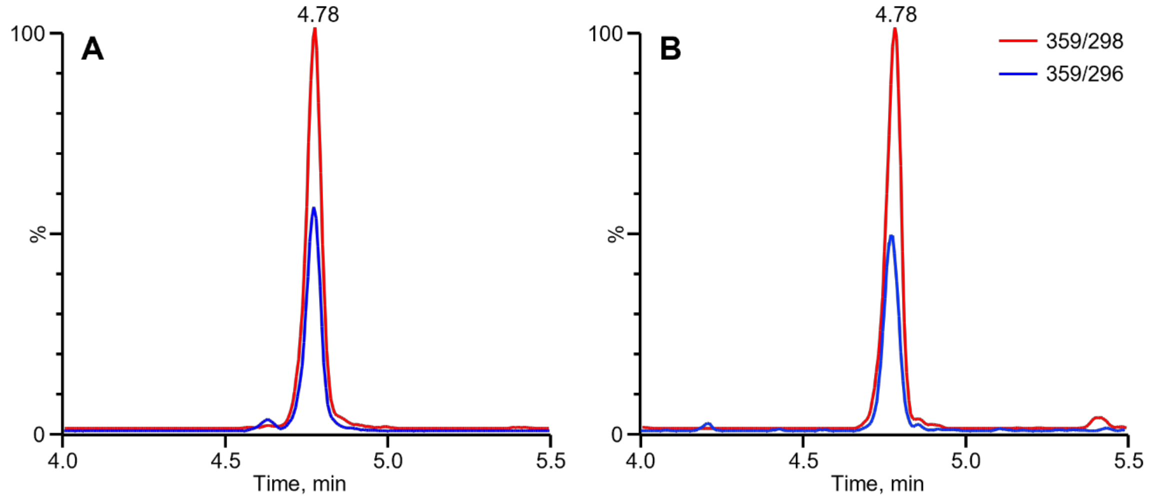

Analysis performed on a Waters TQ-XS triple quadrupole LC-MS/MS system.

Figure S4. Typical LC−MS/MS chromatogram of aristolactam I in (A) standard solution and in (B) an air filter sample.

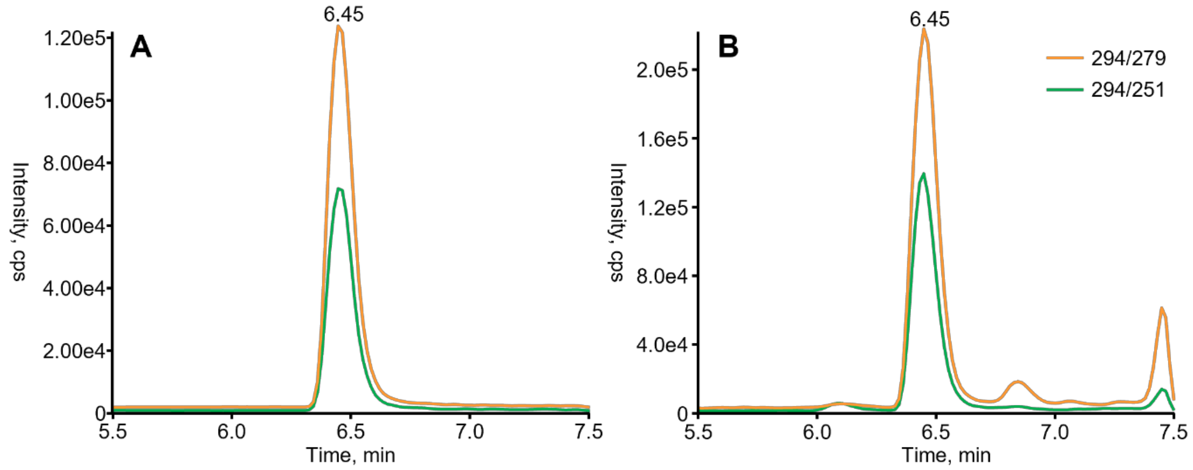

Analysis performed on a Sciex API 4000 QTRAP LC-MS/MS system.

REFERENCES

(1) Chin, M.-L.; Zhang, H.; Au, C.-K.; Luk, W.-L.; Cai, Z.; Chan, W. Aristolochic acids and aristoloxazines are widespread in the soil of Aristolochiaceae herb cultivation fields *Environ. Sci. Technol.* **2024**, 58, 22563–22570.
